# In Vitro and In Vivo Efficacy of Epithelial Barrier-Promoting Barriolides as Potential Therapy for Ulcerative Colitis

**DOI:** 10.3390/biomedicines14010237

**Published:** 2026-01-21

**Authors:** Jon P. Joelsson, Michael J. Parnham, Laurène Froment, Aude Rapet, Andreas Hugi, Janick Stucki, Nina Hobi, Jennifer A. Kricker

**Affiliations:** 1 EpiEndo Pharmaceuticals ehf., Bjargargata 1, 102 Reykjavík, Iceland; 2 AlveoliX AG, Swiss Organs-on-Chip Innovation, 3010 Bern, Switzerland

**Keywords:** Ulcerative colitis, Gut-on-Chip, barriolides, macrolides

## Abstract

**Background/Objectives**: Ulcerative colitis (UC) is an inflammatory bowel disease and a major cause of ulcers and chronic inflammation in the colon and rectum. Recurring symptoms include abdominal pain, rectal bleeding, and diarrhoea, and patients with UC are at a higher risk of developing comorbidities such as colorectal cancer and poor mental health. In UC, the decreased diversity and changed metabolic profile of gut microbiota, along with a diminished mucus layer, leads to disruption of the underlying epithelial barrier, with an ensuing excessive and detrimental inflammatory response. Treatment options currently rely on drugs that reduce the inflammation, but less emphasis has been placed on improving the resilience of the epithelial barrier. Macrolide antibiotics exhibit epithelial barrier-enhancing capacities unrelated to their antibacterial properties. **Methods**: We investigated two novel barriolides, macrolides with reduced antibacterial effects in common bacterial strains. Gut epithelial cell barrier resistance in the Caco-2 cell line, with and without co-culture with mucus-producing HT-29 cells, was increased when treated with barriolides. Using ^AX^Gut-on-Chip technology with inflammatory cytokine-stimulated Caco-2/HT-29 co-cultures, the effectiveness of the barriolides was confirmed. Lastly, we reveal the barrier-enhancing and inflammation-reducing effects of the barriolides in a dextran-sulphate sodium (DSS)-induced colitis mouse model. **Results**: We show the predictive power of the novel ^AX^Gut-on-Chip system and the effectiveness of the novel barriolides. Indications include reduced inflammatory response, increased epithelial barrier and decreased overall clinical score. **Conclusions**: The results of this study indicate the notion that barriolides could be used as a treatment option for UC.

## 1. Introduction

Ulcerative colitis (UC) is a chronic inflammatory bowel disease causing chronic inflammation and ulcers in the colon and rectum, resulting in recurring symptoms, including lower abdominal pain, rectal bleeding, and diarrhoea [1,2]. The incidence of UC continues to rise, and patients are at increased risk of developing comorbidities like colorectal cancer, while their mental health and daily lives are also greatly impacted by the chronicity and unpredictability of UC [3,4]. The pathology of UC is complex, with varying degrees of mucosal inflammation, extending from the rectum to the proximal colon. Risk factors include genetics, environmental factors, autoimmunity and gut microbiota [5]. Under healthy conditions, the epithelial lining cells in the colon act as a protective barrier against injurious insults. Disruption of homeostasis, as in UC, leads to breakdown of the epithelial barrier in the colon, allowing injurious agents and pathogens to enter the tissue and bloodstream, resulting in the development of local inflammation [6].

The only truly effective treatment for UC is surgical removal of the colon, but this is a last resort for therapy-unresponsive patients with severe UC. Most patients are treated with anti-inflammatory drugs to alleviate symptoms and flares [2]. However, existing drugs are only moderately effective, have low safety profiles and benefit only a small fraction of patients [7]. Consequently, there is a major unmet need for drugs with high efficacy and safety.

Many approaches to the development of novel drugs for UC have concentrated on anti-inflammatory and immunosuppressive activity [8]. More recently, research efforts have been directed towards the discovery and development of compounds that act to promote epithelial barrier function. In light of the building evidence, epithelial dysfunction is an early causal event in disease progression rather than a mere consequence of inflammation. Researchers in the USA have provided evidence that inhibitors of myosin light-chain kinase (MLCK) prevent its recruitment under inflammatory stimuli to the perijunctional actomyosin ring at epithelial cell junctions, thereby reducing intestinal barrier loss [9,10]. Although inhibition of MLCK is a promising therapeutic approach, concerns remain regarding long-term safety and potential off-target effects due to the kinase’s broad physiological roles [11]. Alternative strategies, including tight-junction modulators and small molecules, have demonstrated barrier-protective effects in preclinical models [12,13]. However, some of these approaches lack translational validation.

Macrolide antibiotics, including azithromycin, have also been reported to exert immunomodulatory and barrier-associated effects, but their chronic use is limited by antibacterial activity and the associated risk of antimicrobial resistance with chronic use. We have been investigating the epithelial permeability reducing effects of macrolides derived from the antibiotic azithromycin, but which lack antibacterial activity, collectively termed barriolides. EpiEndo Pharmaceuticals developed glasmacinal (EP395), a derivative of azithromycin with negligible antibiotic activity, with clear barrier-protecting properties and activity in various models of neutrophil-dominated lung inflammation that has shown clinical benefit in relation to COPD, confirming the clinical application of the approach taken [14,15]. Based on this success, a series of related compounds was synthesised, and in preliminary screening models similar to those used for EP395, two promising compounds were selected to investigate whether such compounds might exert activity in gut-related pathology, as reported here. Like glasmacinal, these compounds are intended for once-daily, oral dosing.

To test their barrier-enhancing properties we employed complementary in vitro and in vivo models in parallel to optimise prediction accuracy. Cell cultures on inserts are a useful tool for dose finding and to gain insights on a drug’s mechanism of action [16,17], while organ-on-chip technology more closely recapitulates drug safety and efficacy profiles of human patients [18,19] and animal models enable systemic assessment of drug responses [20,21].

Here we describe the properties of two non-antibacterial barriolides, selectively screened for the absence of antibacterial properties and optimised for promotion of epithelial cell barrier function, then subsequently tested for activity in experimental models of UC. IL-8 was selected as an in vivo biomarker of neutrophilic inflammation as it was reproducibly inhibited in inflammatory models responsive to glasmacinal [15].

## 2. Materials and Methods

### 2.1. Antibacterial Susceptibility Testing

Antibacterial activity of barriolides, novel macrolide compounds (EP132 and EP317, EpiEndo Pharmaceuticals, Reykjavik, Iceland), was tested by determining minimal inhibitory concentrations (MICs) using a standard serial broth microdilution technique. The range of test organisms selected were cultured and assayed according to Clinical and Laboratory Standard Guidelines (CLSI) protocols. Inoculum preparation and growth medium of all organisms was cation-adjusted Mueller Hinton +/− lysed horse blood, brucella broth with 7% foetal bovine serum (FBS) for *Helicobacter pylori* or Haemophilus test medium for *Haemophilus influenzae*. Briefly, DMSO stock solutions of the test compounds were diluted in 100% DMSO to generate 6.4 mg/mL working stocks that were 2-fold serially diluted with the same vehicle for a total of 11 titrations. The final bacterial count was 2–8 × 10^5^ colony forming units/mL in a total volume of 200 μL in each well containing 2% DMSO. The final test compound concentrations were 128–0.125 μg/mL. Following incubation, the test plates were examined, and wells scored for growth or complete growth inhibition to define the MIC. Each test substance was evaluated in replicates on the same experimental occasion and tested alongside reference antibiotics for each species of bacteria. The list of microorganisms is shown, alongside the MIC values, in the results in Section 3.1.

### 2.2. Cell Culture

Caco-2 cells (HTB-37, ATCC, Manassas, VA, USA) were cultured in Eagle’s Minimum Essential Medium (EMEM) (30-2003, ATCC, Manassas, VA, USA) with added 20% FBS. HT-29 cells (HTB-38, ATCC) were cultured in McCoy’s 5a Medium Modified (McCoy’s) (30-2007, ATCC, Manassas, VA, USA) with added 10% FBS.

In air–liquid interface culture conditions, the Caco-2 cells were seeded on top of transwell filters and allowed to equilibrate during two days of culturing with media on both the apical and basal sides. After two days, media were removed from the apical compartment, and each subsequent media change was only made in the basal chamber. For liquid–liquid interface (LLI) culture, cells were seeded in the same manner, and all media changes were made both apically and basally. Barriolides and vehicle (up to 0.17% DMSO) were introduced on the basolateral side and refreshed every 2–3 days.

When cells were co-cultured, Caco-2 cells were seeded onto transwell filters in EMEM +20% FBS; then, the following day, HT-29 cells in a 1:9 ratio were seeded on top of the Caco-2 cells in 50% EMEM and 50% McCoy’s with 10% FBS with media in the apical and basal chambers of the transwell setup (LLI).

#### 2.2.1. Transepithelial Electrical Resistance (TEER)

Transepithelial electrical resistance (TEER) of cell layers on transwell filters was measured using a Millicell ERS-2 Epithelial Volt-OHM Meter (MER00002, Millipore, Burlington, MA, USA) with chopstick electrodes (MERSSTX01, Millicell ERS Probes, Millipore, Burlington, MA, USA). Chopsticks were sterilised and one point was inserted into the media in the well, while the other point was inserted into the media on top of the transwell filter. This allowed for electrical resistance to be measured.

#### 2.2.2. Imaging

Cell layers on transwell filters were fixed in formalin (10% buffered formaldehyde) and embedded in paraffin, sectioned and immunostained with haematoxylin and eosin. Samples were imaged using a Leica DM500 with added Leica ICC50W (Leica Microsystems, Wetzlar, Germany).

For transmission electron microscopy, cell layers were fixed in 2.5% glutaraldehyde for 30 min. This was followed by fixation on coverslips, which were placed in 2% osmium tetroxide, and subsequently rinsed in phosphate buffer. Cell layers were dehydrated and embedded in resin. Sections were cut with an Ultramicrotome (Leica EM UC7, Leica Microsystems, Wetzlar, Germany) and stained with lead citrate (3%, J.T. Baker Chemical Co., Phillipsburg, NJ, USA). Samples were then imaged using a JEM-1400PLUS PL Transmission Electron Microscope (JEOL Ltd., Tokyo, Japan).

#### 2.2.3. Viability Studies

For viability studies, compounds were dispensed into a 384-well black bottom plate (3571, Corning, NYC, NY, USA). Cells were prepared (100.000/mL, 25 μL per well) and dispensed into wells containing compounds. CellTiter-Glo Luminescent Cell Viability Assay (G7570, Promega, Madison, WI, USA) solution was added with a dispenser (20 μL per well). The signal was read with a plate reader (2114 Multilabel Reader, PerkinElmer, Waltham, MA, USA).

### 2.3. ^AX^Gut-on-Chip

#### 2.3.1. ^AX^Barrier-on-Chip System

**Figure 1:**
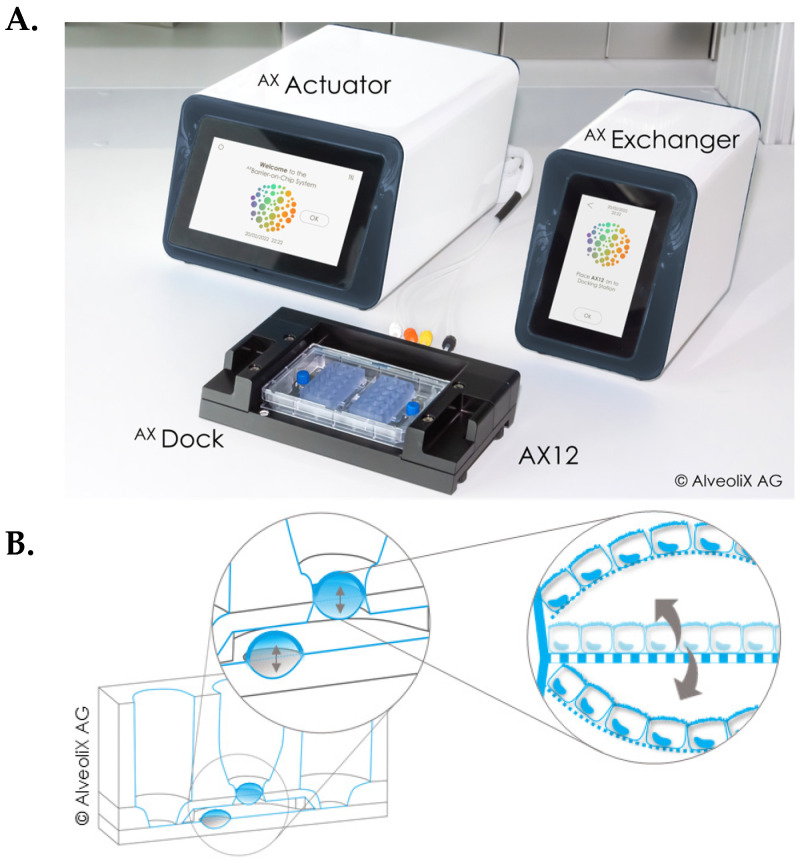
Overview of the ^AX^Barrier-on-Chip technology. (**A**) It includes a microfluidic chip, called AX12, two electropneumatic modules, the ^AX^Actuator and ^AX^Exchanger and an interface platform, ^AX^Dock. (**B**) Principle of bi-directional 3D-stretch. At the bottom of the cell compartment, the ^AX^Actuator can cyclically pull a membrane up and down (indicated by arrows). This deflection is transferred to the porous membrane, where the cells are cultured.

The ^AX^Barrier-on-Chip system [22] (AlveoliX, Bern, Switzerland) includes the AX12 plate consumable, two electropneumatic devices (the ^AX^Exchanger and ^AX^Actuator) and an interface unit (^AX^Dock). The ^AX^Actuator enables precise adjustment of 3D-stretch parameters, allowing for customisation of strain frequency, amplitude, direction (uni- or bi-directional) and duration to meet specific experimental requirements (Figure 1).

#### 2.3.2. ^AX^Gut-on-Chip Injury Model

The human colon adenocarcinoma epithelial cell line Caco-2 (86010202, ECACC, Salisbury, UK) was cultured in AX Intestinal Epithelial Medium (^AX^IEM, AlveoliX, Bern, Switzerland). The human colon cell line HT-29 (91072201, ECACC, Salisbury, UK) was cultured in AX Intestinal Secretory Cell Medium (^AX^ISCM, AlveoliX, Bern, Switzerland). For co-culture, Caco-2 and HT-29 cells were seeded sequentially on the apical side of the membrane at a 9:1 ratio. The medium was refreshed every 2–3 days, with AX Intestinal Epithelial Barrier Medium (^AX^IEBM, AlveoliX, Bern, Switzerland) used for the co-culture. Mechanical unidirectional strain mimicking the natural digestive contractions of the intestinal wall was applied with cyclic deflection of the membranes for 16 h followed by 8 h of rest. Upon barrier formation and stabilisation, barriolides and vehicle were applied 10 days prior to cytokine challenge (injury). Cell culture medium containing 0.17% DMSO (BioLife Solutions, Bothell, WA, USA) was used as vehicle. Barriolides and vehicle were introduced through the apical and basolateral sides and refreshed every 2–3 days. Barriolide effects on barrier development were evaluated using TEER measurement over 10 days.

The molecules were incubated for a further 48 h in the injury setting. Injury was induced on Day 14 using a cytokine cocktail of 20 ng/mL TNFα (300-01A, PeproTech, Rocky Hill, NJ, USA), 10 ng/mL INFγ (300-02, PeproTech, Rocky Hill, NJ, USA) and 10 ng/mL IL-1β (200-01B, PeproTech, Rocky Hill, NJ, USA). The cytokine cocktail was added in cell culture medium to the apical and basolateral sides, and cells were incubated for an additional 48 h in the presence of the vehicle or test molecules.

#### 2.3.3. TEER Measurement and Analysis

TEER data values were recorded at different timepoints using the commercial TEER measurement device provided by AlveoliX AG (Bern, Switzerland). The background value was subtracted (empty well). Resistance values were then referred to the membrane area and presented in Ω∙cm^2^. To compare trends among treatments, values are also shown relative to the vehicle group average.

#### 2.3.4. Cytotoxicity Analysis

Cytotoxicity was assessed using the LDH-Glo Cytotoxicity Assay (J2380, Promega, Madison, WI, USA). The supernatant was sampled at the endpoint and preserved according to the manufacturer’s instructions. To normalise the two rounds and compare trends among treatments, values are shown relative to the vehicle group average.

#### 2.3.5. Cytokine Analysis

Medium supernatant was sampled apically at the endpoint and stored at −80 °C. All samples were analysed using the Human IL-8/CXCL8 DuoSet ELISA (DY208, R&D Systems, Minneapolis, MN, USA). Values are shown relative to the injury group average.

### 2.4. Mouse Dextran-Sulphate Sodium-Induced Colitis Model

#### 2.4.1. Animal Ethics Statement

Animal treatment, care and experiments were performed in accordance with the authority of the UK Home Office Procedural Project Licence PEEC6BC38, and under the UK Animal (Scientific Procedures) Act 1986, amended in 2012, and the ARRIVE guidelines. The animal facilities have full AAALAC accreditation, and the study followed the facilities’ standard operating procedures.

#### 2.4.2. Mouse Model of Ulcerative Colitis

Adult male C57BL/6 mice from Charles Rivers Laboratories (Portishead, UK) had an average weight of 21.8 g (18.7 to 26.3 g) upon arrival and were randomised into five experimental groups based on their bodyweights and to ensure each group had a similar mean bodyweight. Group sizing was based upon historical data. Animals were allowed to acclimatise for one week and then maintained in standard animal housing consisting of a 12 h light dark cycle with dawn and dusk phases. Room temperature and humidity were maintained within standard environment guidelines (20–24 °C and 45–65%, respectively). Environmental enrichment and ad libitum chow and water were provided in all cages throughout the study.

Under all conditions (2.5% DMSO vehicle, EP317, EP132, and cyclosporin A with DSS) *n* = 10 animals, except the vehicle control group, (absence of DSS), where *n* = 5. Barriolide treatments (5 mg/kg) were administered per os (p.o.) once daily for 21 days from day 0. On day 14, the reference compound, cyclosporin A (Neoral^®^, Novartis, Basel, Switzerland) oral solution was diluted in DPBS to a concentration of 7.5 mg/mL and was administered p.o. once a day for 7 days. Also on day 14, animals were given ad libitum access to 3% dextran sulphate sodium (DSS) in water until the end of the study to induce a mouse model of ulcerative colitis. Bodyweight was monitored throughout the study, and clinical scores were assessed on days 14–20, including blood in stool and stool consistency. Samples for lipocalin-2 and cytokine assays were collected at Day 20 to assess overall well-being, blood in stool and stool consistency. On Day 21, animals were anaesthetised by 5% gaseous isoflurane and bled via their orbital sinus vein. Immediately after, they were terminated via cervical dislocation. Colons were dissected for gross pathology and distal colon explant cultures were set up and 48 h supernatants were stored for Luminex cytokine analysis. Also on Day 21, faecal samples were frozen for lipocalin-2 quantification by ELISA (R&D Systems, DY1857, Minneapolis, MN, USA) as per the manufacturer’s instructions.

#### 2.4.3. Explant Cell Cultures

At necropsy, colons were removed and washed three times. Longitudinal sections were taken and weighed. Explant cultures were set up in DMEM, and after 48 h, super-natants were stored for analysis by Luminex. Samples were analysed in singlicate and run neat, with each point representing the result for a single animal.

### 2.5. Statistical Analysis

Graphs were generated in GraphPad Prism v.10.6.1 (GraphPad Software, San Diego, CA, USA). Statistical significance in cell culture models was determined using one-or two-way ANOVA, depending on the appropriateness. Additional multiple comparison tests were performed as required. In vitro cell culture data as was analysed using Dunnett’s multiple comparison test. Statistical significance of mouse clinical data was analysed by ordinary one-way ANOVA followed by Sidak’s multiple comparisons test, the mean of the vehicle control group was compared against the mean of the vehicle to ensure model efficiency, and then the mean of the vehicle was compared against the mean of all other groups. Mouse cytokine data statistical significance was determined using Kruskal–Wallis followed by Dunn’s multiple comparison test. * *p* < 0.05, ** *p* < 0.01, *** *p* < 0.001

## 3. Results

### 3.1. Antibacterial Activity of Barriolides Is Minimal

We generated novel macrolide compounds with the aim of minimising antibacterial activity while retaining epithelial barrier-enhancing properties. We selected a range of common Gram negative and positive microorganisms to determine MIC values of EP317 and EP132 using an in vitro microdilution assay. Using standard antimicrobial testing across a conventional concentration range (up to 128 µg/mL), compounds were tested in duplicate against a set of reference compounds. The microorganisms and MIC values are presented in Table 1. Both compounds exhibited substantially higher MIC values than effective antibiotic comparators, indicating minimal antibacterial potency. For the most sensitive species tested (*Moraxella catarrhalis*), EP317 and EP132 were approximately 64-fold and 128-fold less potent than the reference compound ciprofloxacin, respectively. These findings support the classification of these compounds as having diminished antibiotic activity. Azithromycin was not included in this assay because the most effective antibiotic was used as the comparator; comparative MIC data with azithromycin for a related compound (EP395) have been reported previously [14].

### 3.2. Liquid–Liquid Interface Induces Polarised Epithelium

Finding the appropriate conditions for culturing gut epithelial cells is critical for the proper in vivo representation of the human gut epithelium. For this reason, we tested the Caco-2 cell line in both air–liquid (ALI) and liquid–liquid interface (LLI) conditions on transwell inserts. AZM, a macrolide known to enhance the epithelial barrier [23,24,25,26], was used as a reference compound.

**Figure 2:**
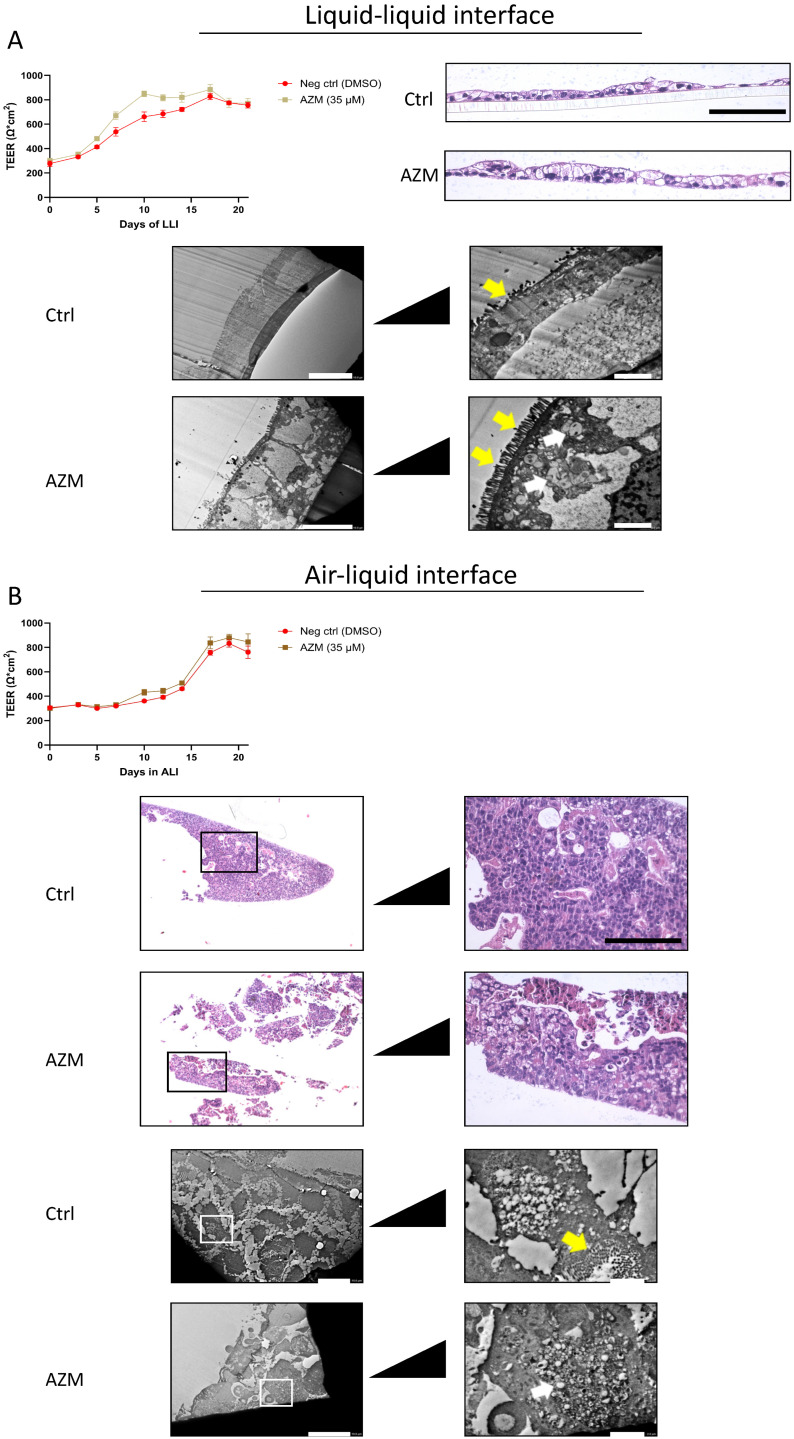
Comparison of barrier formation and polarisation under LLI and ALI culture conditions. (**A**) Caco-2 cells were cultured under LLI conditions. AZM treatment did not affect the rise in TEER as both culture conditions generated the same level of resistance after 3 weeks. Cross-sectional imaging revealed a slightly thicker cell layer in the AZM-treated cells, and TEM images show a well-polarised cell layer. (**B**) Cells that were cultured in ALI conditions provided no indication that AZM affected TEER better than control. Histologically, the cells did not appear polarised with cell aggregates throughout the cell layer. TEER values (Ω∙cm^2^) are presented as average ± SD. Scale bars for histological images are 50 µm and scale bars for TEM images are 2 and 10 µm, respectively. Yellow arrows point to microvilli; white arrows point to vesicles.

TEER measurements of LLI cultures indicated no significant increase with AZM treatment. Viewing cross-sectional images of the gut epithelium, however, showed indications of a slightly thicker cell layer, with increased vesicular formations (Figure 2A). Electron microscope images clearly indicate a well polarised cell layer, where microvilli are visible on the apical side. Antithetically, ALI-cultured cells showed no clear polarisation of the cells, with microvilli formation inside the cell layer (Figure 2B). Cross-sections of the cell layer indicate a much thicker aggregation of cells, where cells are growing on top of each other. This, however, did not result in higher TEER readings.

### 3.3. Barriolides Increase Barrier Strength of Gut Epithelium In Vitro

**Figure 3:**
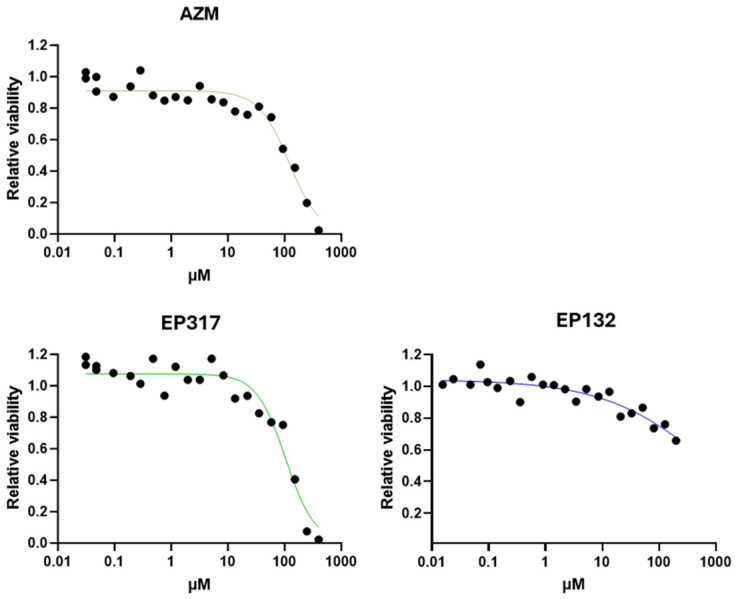
Caco-2 cell viability. Caco-2 cell viability was tested using AZM, EP317, and EP132. Cell viability decreased at higher concentrations in a similar fashion for all compounds, although EP132 shows a rather milder effect at higher dose ranges. Increasing concentrations correlate with increasing colour depth.

**Figure 4:**
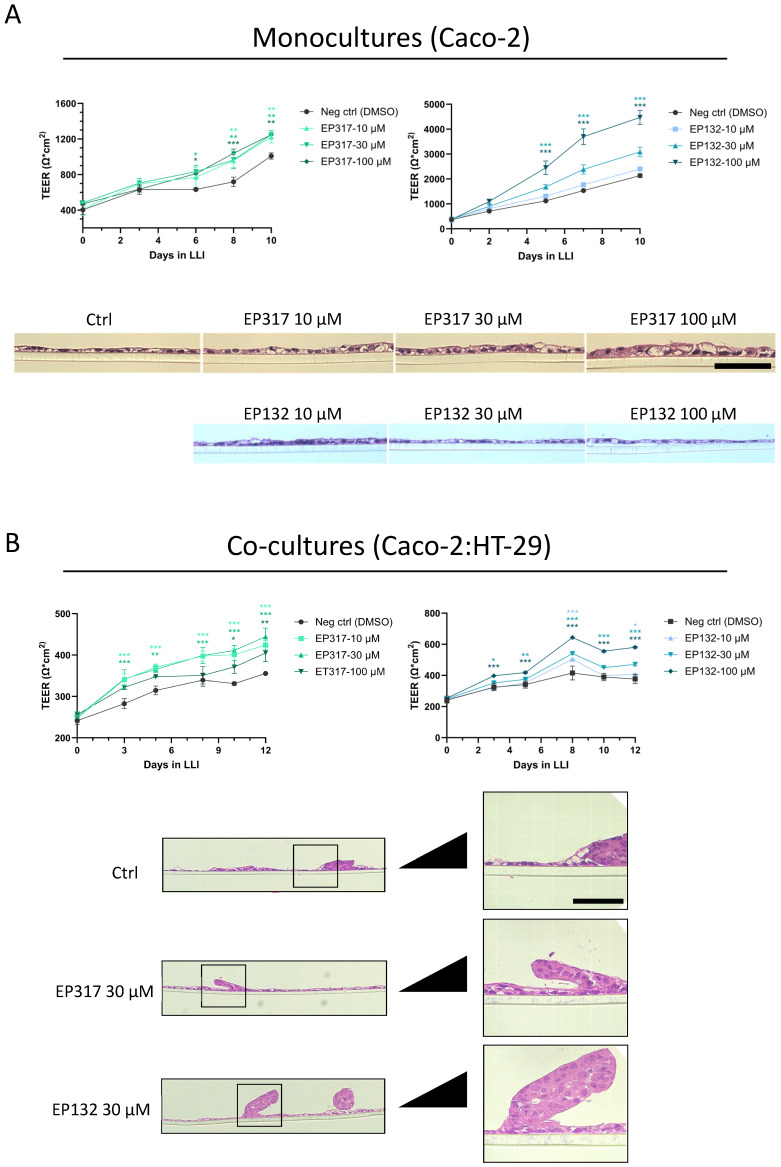
Barrier integrity in Caco-2 and Caco-2/HT-29 co-cultures. (**A**) Treating Caco-2 cells cultured on transwell inserts with either EP317 or EP132 resulted in significantly higher TEER readings over ten days. EP132 increased TEER in a concentration-dependent manner, while EP317 increased TEER independently of the concentrations tested. Cross-sectional images revealed a slight thickening of the cell layer in cells treated with EP317, which was not observed for EP132-treated cells. (**B**) Co-culturing Caco-2 cells with HT-29 cells at a 9:1 ratio resulted in lower TEER values. The test compounds exerted similar effects on TEER values. Cross-sectional images reveal an aggregation of cells on top of the cell layer. Scale bars for histological images are 50 µm. Statistical significance in cell culture models was determined using two-way ANOVA, followed by Dunnett’s multiple comparison test. * *p* < 0.05, ** *p* < 0.01, *** *p* < 0.001.

Because of the findings of the LLI and ALI comparisons, the two barriolides, EP317 and EP132, were tested in the established LLI cell model. Cell viability of EP317 was comparable to that of AZM, with relative IC50 values of 76.2 and 71.1 µM, respectively. EP132 reduced cell viability by only 34% at the top concentration of 400 µM; thus, an IC50 was not calculated (Figure 3). Both barriolide treatments resulted in significantly higher TEER than in the control cell layers (Figure 4A), with EP132 increasing TEER in a concentration-dependent manner. Cross-sectional images revealed a thickening of the cell layer in EP317-treated cells, especially at higher concentrations. This was not seen in EP132-treated cells.

As TEER rose to relatively high levels, it became apparent that this tight epithelium did not represent the gut epithelium adequately, and thus Caco-2 cells were co-cultured with another colonic mucus-producing epithelial cell line, HT-29. This resulted in lowering of the TEER, evidently mimicking more the permeable barrier and varied cells of the gut epithelium, and forming a more in vivo-like gut epithelial cell layer. The effect of the barriolide treatment was retained and consistent with the results obtained in monoculture in increasing barrier function in the co-cultures (Figure 4B). Histology showed aggregations of cells at various sites on the cell layer when cells were co-cultured, consistent with more organ-like structures (Figure 4B).

### 3.4. EP132 Treatment Showed Increased Efficacy in the ^AX^Gut-on-Chip Model

**Figure 5:**
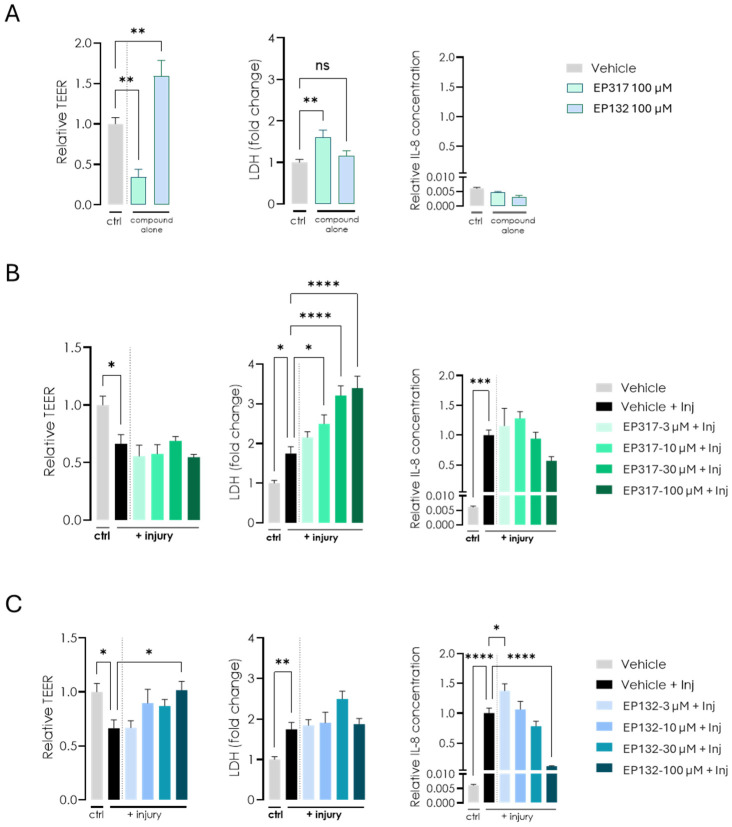
Effects of EP132 and EP317 on barrier function in the ^AX^Gut-on-Chip model. (**A**) Cells were cultured in the ^AX^Gut-on-Chip model, where only EP132 treatment resulted in increased TEER. EP317 treatment resulted in slightly higher cell death, as indicated by increased LDH levels. IL-8 levels were unchanged for both treatments. (**B**,**C**) The cytokine cocktail (IFNγ, TNFα and IL-1β) introduced to the cell cultures resulted in decreased TEER. EP132 treatment only ameliorated this effect at high doses. Cell death was unaffected by EP132 treatment, while the increase in IL-8 induced by the injurious cytokine cocktail was reduced by EP132 treatment of the cells. Statistical significance in the ^AX^Gut-on-Chip model was determined using one-way ANOVA, followed by Dunnett’s multiple comparison test: ns: not significant, * *p* < 0.05, ** *p* < 0.01, *** *p* < 0.001, **** *p* < 0.0001.

Transwell cultures under LLI conditions are a standard in vitro model but have limitations due to their simplicity and static environment. Thus, we decided to test our barriolides in a system that includes the in vivo dynamic setting of the colon: the ^AX^Barrier-on-Chip system. Initial in vitro on-chip studies with the compounds were performed using a high concentration (100 μM) to maximise the probability of detecting a response on TEER in this higher complexity system, consistent with a screening approach prior to dose–response refinement. This concentration is within the same magnitude of earlier studies using AZM [27,28]. EP317 treatment at this high concentration resulted in the lowering of TEER, similar to AZM, which was associated with cell death, as revealed via increased LDH levels. EP132 at the same concentration did not negatively affect cell viability and resulted in increased TEER, similar to what was observed on transwell inserts. A panel of epithelial-derived inflammatory readouts were measured in this immune cell-free system (Supplementary Materials Figure S1); however, IL-8 was the most sensitive and reproducible. IL-6 protein levels were also increased following the injury cocktail stimulus, and EP132 showed a trend towards reducing this increase, although these findings require confirmation with additional independent experiments. IL-8 protein levels were unchanged for both treatments, although a trend towards lowered expression was observed (Figure 5A). Using a cytokine cocktail of IFNγ, TNFα and IL-1β as an injurious inflammatory stimulus, TEER was reduced in the cell cultures. EP317 treatment did not prevent this lowered TEER, regardless of concentration, possibly due to the increased cell death at higher concentrations. Generation of IL-8 was not significantly affected by EP317 treatment in this setup (Figure 5B). In contrast, EP132 treatment ameliorated the induced decrease in TEER caused by the inflammatory cocktail but had no impact on the increased cell death. Its effect was also demonstrated by the generation of IL-8 upon the inflammatory cocktail stimulus, which decreased with increasing concentrations of EP132 (Figure 5C).

### 3.5. Barriolides Protect from Injury in a DSS Model of UC

**Figure 6:**
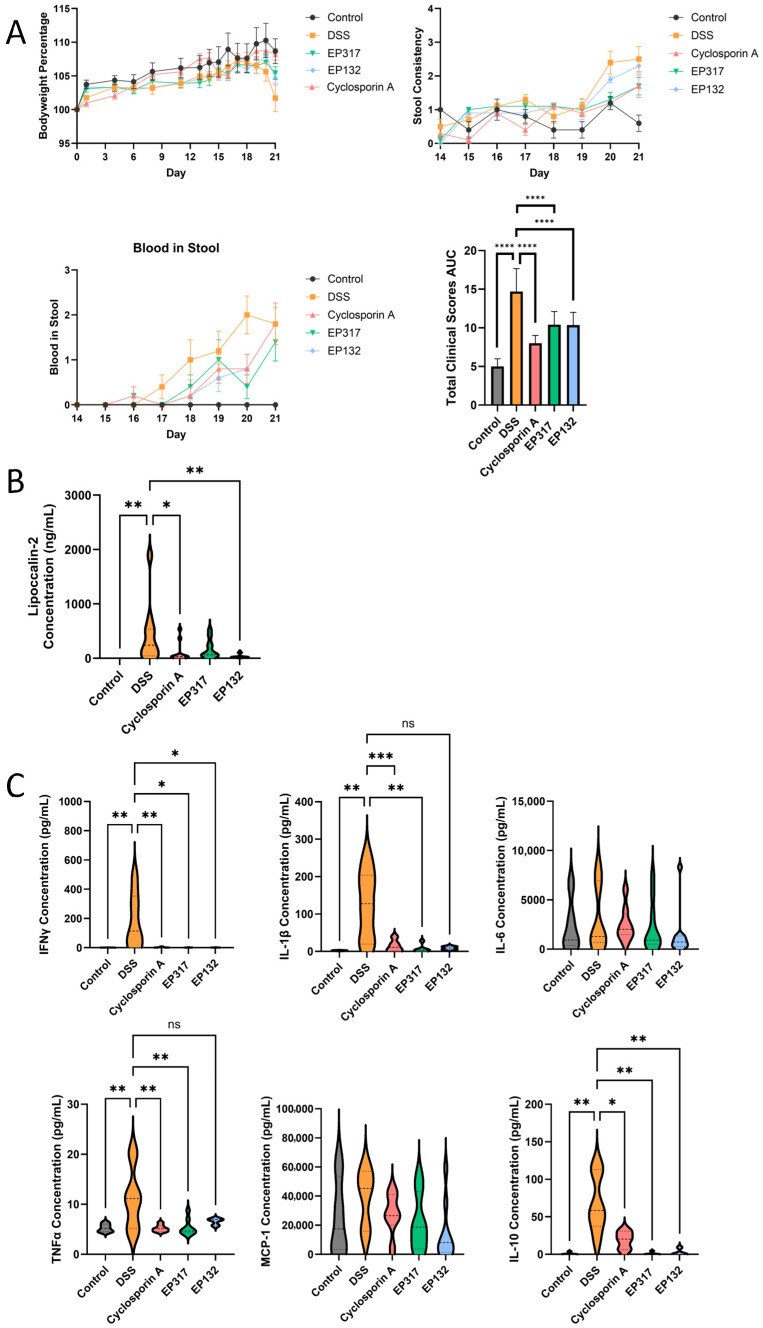
Effects of barriolide treatment in a DSS-induced colitis model. (**A**) Individual clinical scores and the total clinical score (bodyweight loss, loose stools and presence of gross blood in the stools) as AUC from day 14–21 of 3% DSS-induced injury in mice pre-treated with barriolides and control group mice. Data are presented as total score ± SEM. (**B**) Faecal samples were collected, cells lysed and supernatants measured for lipocalin-2, a marker of intestinal inflammation. EP132 treatment of the mice resulted in lower levels of lipocalin-2. (**C**) On Day 21 colons were dissected, and distal colon explant cultures were set up. After 48 h, supernatants were collected and frozen for cytokine analysis by Luminex. Inflammatory cytokine concentrations were determined in colon explants. Barriolide pre-treatments were found to attenuate the induced expression of IFNγ, IL-1β, TNFα and IL-10. Data shown in panels (**B**,**C**) are presented as violin plots of concentrations from individual animals in each group, whereby the plot shows the distribution of the data points. Some samples were below the lower level of quantification. *n* = 5 vehicle control; *n* = 10 DSS-treated mice. Statistical significance of mouse clinical data was analysed by ordinary one-way ANOVA followed by Sidak’s multiple comparisons test, the mean of the vehicle control group was compared against the mean of the vehicle to ensure model efficiency, and then the mean of the vehicle was compared against the mean of all other groups. Mouse cytokine data statistical significance was determined using Kruskal–-Wallis followed by Dunn’s multiple comparison test: ns: not significant, * *p* < 0.05, ** *p* < 0.01, *** *p* < 0.001, **** *p* < 0.0001.

The same barriolides were tested in an established mouse model of ulcerative colitis, where mice receive 3% DSS in drinking water daily for a week to induce a moderate disease state. Figure 6A shows that bodyweight started to decrease in mice that received DSS, and this effect was somewhat ameliorated in mice that received barriolides. Stool consistency was comparable between treatments and the positive control, cyclosporin A. Compared to vehicle control mice, all active treatments resulted in a delayed onset of blood in stool for the mice. The total clinical scores (averaged bodyweight loss, loose stools and/or diarrhoea and presence of occult or gross blood in the stools) of mice with DSS injury were diminished by both barriolides to a level comparable to that of the positive control (Figure 6A). Faecal lipocalin-2, a marker of intestinal inflammation, was measured to assess both the extent of the DSS-induced injury and barriolide efficacy. Levels of lipocalin-2 in the concentrated faecal samples taken on day 21 were attenuated significantly by EP132 pre-treatment (Figure 6B). Although EP317 also tended to reduce lipocalin-2 levels, a significant difference from the untreated DSS mice was not achieved. Among cytokines assayed in distal colon explant supernatants, DSS injury induced expression of IFNγ, IL-1β, TNFα and IL-10. This effect was attenuated by both barriolide pre-treatments. IL-6 and MCP-1 levels were unaffected by barriolide treatment (Figure 6C).

## 4. Discussion

During the last two to three decades, researchers have unveiled much of the pathogenesis and aetiology of UC. This has led to advances in treatment options. Still, current treatment options for UC are limited to targeting the inflammatory effects of the disease, while the underlying cause of intestinal barrier failure has received less attention [7]. In this study, we describe the barrier-enhancing properties of novel barriolides. This was performed progressively with in vitro methods by first treating gut cells with barriolides, introducing cyclical motions which mimic the biomechanic forces of the colon, before recreating injury with an inflammatory cytokine challenge. Subsequently, the compounds were tested in vivo in the well-established murine model of colitis induced by DSS.

Selecting the appropriate cell culture model to mimic the human condition can be extremely complex. When the system is too simple, the results may lack relevance when applied to the immense complexity of the human body. On the other hand, when the cell culture model is too complex, there are many confounding variables that could skew the results. We have approached this challenge by enhancing complexity in a stepwise manner. We started by comparing known cell culture methods for epithelial cells, ALI vs. LLI. We found that gut epithelial cells, when cultured in ALI, tended to grow uncontrollably and the cells did not assemble in a polarised cell layer, but when cultured in LLI, the cell formed a polarised epithelium (Figure 2A,B). Relying on the LLI condition, we initiated our studies using the two barriolides specifically designed for this study. Both barriolides, particularly EP132, increased TEER in Caco-2 cells (Figure 4A).

A concern around mimicking the highly permeable gut epithelium was that exceedingly high TEER values would decrease the appropriateness of using the Caco-2 cells; therefore, we started co-culturing Caco-2 with HT-29 intestinal cells [29,30]. This resulted in lower overall TEER of the cell layer, while the clear barrier-enhancing effects of the barriolides were still pronounced (Figure 4B). When cultured alone, the EP317 barriolide treatment of the Caco-2 cells resulted in a slightly thicker cell layer at the highest concentrations tested. This could be due to increased lipid vesicle formation, as reported for lung epithelial cells that have been treated with the macrolide AZM [23]. This apparent increase in the thickness of the cell layer was not seen in the EP132-treated cells. This could be due to the inherent differences in the compounds themselves. A logical next step would be to investigate whether the compounds affect lipid vesicle formation in vivo. In co-culture, the cell layer appeared similar to that in monocultures, except for aggregates of cells occurring throughout the cell layer (Figure 4). It could be that the decrease in TEER is a result of these aggregations, which are a common occurrence in co-cultures, reflecting organ-like phenomena. Similar histological observations in Caco-2/HT-29 co-cultures have been reported [30,31].

AlveoliX developed a platform allowing us to mimic the dynamic microenvironment of several organs including the intestines. Such human-relevant models are expected to gain more importance in the future with the release of new regulations such as the FDA NIH modernization act 3.0 that promote the replacement of pre-clinical animal models for specific drug categories [32]. Several studies have successfully integrated microphysiological systems (MPS) data into their pre-clinical drug development pipeline for safety and efficacy testing [33,34]. However, the limited number of parallel studies comparing in vivo and in vitro results is a major hurdle for broad acceptance of MPS by regulatory bodies [35]. This issue has been addressed in this study by designing drug testing protocols in advanced in vitro models that translate to the DSS-induced mouse model of UC. When setting up the ^AX^Gut-on-Chip, initial studies involved measuring TEER in co-cultured Caco-2/HT-29 cells at relatively high concentrations of barriolides, based on initial screening studies. These studies indicated that TEER was increased when cells were pre-treated with EP132 and cell death was not affected, but EP317 pre-treatment had the opposite effect, increased cell death, and as a result, the TEER was decreased (Figure 5A). The higher rate of cell death was seen even at low concentrations of EP317 (Figure 5B), with an IC50 similar to that of AZM. TEER reduction induced with a cocktail of cytokines was ameliorated by EP132 pre-treatment of the cells, even at lower concentrations, and the effect of the EP132 treatment on cell death was not as pronounced as with EP317. Cytokine cocktail-induced IL-8 generation was also reduced by a high concentration of EP132 pre-treatment, reflecting anti-inflammatory activity (Figure 5C) subsequent to the barrier injury.

We next endeavoured to translate the in vitro data to the in vivo situation using the murine DSS-induced colitis model, in which the animals were pre-treated with barriolides or cyclosporin. Loss of bodyweight, stool consistency and blood in stool were all beneficially affected by treatment with the compounds, resulting in lower total clinical scores in animals that had received barriolide treatment (Figure 6A). The DSS injury model was shown to be active due to the increases in the inflammatory response and lipocalin expression. Barriolide treatment showed comparable beneficial inhibitory effects to the positive control (Cyclosporin A) (Figure 5B,C). Some of the samples from the treatment group animals were below the lower limit of quantifications, indicating that the treatments effectively reduced the DSS-induced elevated cytokine concentrations.

Although direct comparisons between the ^AX^Gut-on-Chip and the in vivo model are limited by their differing endpoints, the data from the ^AX^Gut-on-Chip injury model align with the conclusions drawn from the DSS model. Both models indicate that the barriolides can ameliorate induced injury. Additional endpoints—such as the inclusion of reproducible and comparable cytokine measurements—would help to further evaluate the similarities between the two systems. However, inherent differences between in vitro and in vivo models, including the specific markers that can be assessed in each, may remain a limiting factor.

Together, the ^AX^Gut-on-Chip and DSS colitis data demonstrate that the advanced in vitro model has predictive value for in vivo efficacy of barriolides and that the two approaches are highly complementary. In the ^AX^Gut-on-Chip model, EP132 strengthened epithelial barrier function under both basal and cytokine-injured conditions, as reflected by sustained TEER and reduced IL-8 release, whereas EP317 was limited by increased cytotoxicity and failure to preserve TEER. This profile was mirrored in vivo, where both compounds improved clinical scores, but only EP132 significantly reduced faecal lipocalin-2 and consistently attenuated pro-inflammatory cytokines in distal colon explants. Thus, the chip platform not only anticipated the barrier-protective activity of barriolides but also predicted the therapeutic window of EP132 relative to EP317. More broadly, the sequential use of transwell cultures, the ^AX^Gut-on-Chip model and the DSS mouse model illustrates how mechanistic, human-based in vitro data can guide compound selection.

Other strategies of UC drug development rely on boosting the epithelium, with the goal to counter any disruption in the physical integrity of the barrier which would allow pathogens to invade and cause mucosal dysbiosis, ultimately disturbing gut homeostasis and potentially leading to conditions such as inflammatory bowel disease. Recently, increased research effort has been put into enhancing the intestinal epithelial barrier. A promising epithelial barrier-promoting drug, divertin, was recently introduced, whereby the drug corrects the epithelial dysfunction by blocking TNF-induced MLCK1 recruitment, along with downstream MLC phosphorylation [10]. Other drugs, such as budesonide [36] and glucocorticoids [37], have been introduced as barrier-enhancing drugs, albeit in the airway epithelium. Although macrolides are not commonly associated with treatment of inflammatory bowel diseases, a recent study by Elkholy et al. showed that a daily oral dose of AZM significantly reduced DSS-induced clinical scores in rats [27], similar to our findings with the barriolides shown here. Others have also explored the potential benefits of AZM with and without metronidazole, an antibiotic commonly prescribed in UC treatment, either in animal studies [38] or in a systematic review of paediatric patients [39], with positive conclusions. Importantly, barriolides differ fundamentally from azithromycin in that they were designed to retain beneficial non-antibiotic properties while having negligible antibacterial activity. This design strategy aimed to preserve the beneficial barrier-protective and immune-modulating effects, while mitigating the risk of antimicrobial resistance, impactful microbiome disruption and off-target effects linked to chronic antibiotic use.

In vitro and in vivo evidence presented here indicates that both tested barriolides show significant efficacy in increasing barrier integrity and dampening the cytokines associated downstream from an epithelial injury-induced immune response. Across the three models of evaluation, EP132 showed clear dose-dependent effects in barrier strengthening in co-cultures while remaining non-toxic at higher concentrations, resulted in significant reductions in cytokines in response to injury in the ^AX^Gut-on-Chip model and had beneficial impacts in the mouse DSS model. While EP317 also showed promising attributes, EP132 performed consistently across all evaluations, and appears to be the superior candidate drug in the inflamed gut. Further studies are needed to determine the relative contributions of epithelial barrier protection and direct anti-inflammatory effects towards the beneficial effects of the compounds in DSS colitis in vivo. However, together, the data are encouraging for the further development of barriolides for the treatment of colitis.

## 5. Conclusions

We have established a progressive testing system to evaluate compounds acting on intestinal epithelial barrier function and identified barriolides as effective in this respect. Additionally, we have identified EP132 as a promising compound for further investigation.

## Figures and Tables

**Table 1:** MIC values demonstrate diminished antibiotic activity of EP317 and EP132.

		MIC µg/mL		
Species	Isolate	EP317	EP132	Reference Cpd
*Bacillus subtilis*	ATCC 43223	>128	>128	Vancomycin 0.25
*Enterococcus faecalis*, VanA	ATCC 29212	>128	>128	Vancomycin 2.0
*Enterococcus faecium*	TUH44-29, CCUG 59167	>128	>128	Linezolid 4.0
*Escherichia coli*	ATCC 10536	128	128	Ciprofloxacin 0.0078
*Escherichia coli*	ATCC 25922	>128	>128	
*Haemophilus influenzae*	ATCC 35056	64	128	Ciprofloxacin 0.0156
*Helicobacter pylori*	ATCC 43504	32	>128	Tetracycline 0.5
*Klebsiella pneumoniae*	ATCC 43816	>128	>128	Ciprofloxacin 0.031
*Moraxella catarrhalis*	ATCC 25238	4	8	Ciprofloxacin 0.063
*Pseudomonas aeruginosa*	ATCC 27853	>128	>128	Ciprofloxacin 0.25
*Staphylococcus aureus*, MRSA	ATCC 33591	>128	>128	Vancomycin 1.0
*Staphylococcus aureus*	ATCC 29213	>128	>128	
*Streptococcus pneumoniae*	ATCC 49619	64	>128	Vancomycin 0.25
*Streptococcus pyogenes*	ATCC 14289	64	>128	Vancomycin 0.5

## Data Availability

The data presented in this study are available on request from the corresponding author. Due to sensitive information involving barriolide compounds, the availability of these data is somewhat limited.

## Supplementary Materials

The following supporting information can be downloaded at https://www.mdpi.com/article/10.3390/biomedicines14010237/s1, Figure S1: EP132 and EP317 differentially regulate cytokine release after epithelial injury in ^AX^Gut-on-Chip.

## Abbreviations

The following abbreviations are used in this manuscript:

ALI	Air–liquid interface
AZM	Azithromycin
DSS	Dextran-sulphate sodium
LLI	Liquid–liquid interface
TEER	Transepithelial electrical resistance
UC	Ulcerative colitis

## References

[1] Du L., Ha C. (2020). Epidemiology and Pathogenesis of Ulcerative Colitis. Gastroenterol. Clin. N. Am..

[2] Le Berre C., Honap S., Peyrin-Biroulet L. (2023). Ulcerative colitis. Lancet.

[3] Everhov A.H., Askling J., Soderling J., Halfvarson J., Eriksson J., Smedby K.E., Ludvigsson J.F., Sorensen H.T., Olen O. (2025). Cancer incidence in patients with ulcerative colitis naive to or treated with thiopurine and targeted therapies-a cohort study 2007 to 2022 with comparison to the general population. J. Crohn’s Colitis.

[4] Yan T., Su T., Zhu M., Qing Q., Huang B., Liu J., Ma T. (2025). Oxidative stress gene expression in ulcerative colitis: Implications for colon cancer biomarker discovery. Sci. Rep..

[5] Gajendran M., Loganathan P., Jimenez G., Catinella A.P., Ng N., Umapathy C., Ziade N., Hashash J.G. (2019). A comprehensive review and update on ulcerative colitis. Dis. Mon..

[6] Odenwald M.A., Turner J.R. (2017). The intestinal epithelial barrier: A therapeutic target?. Nat. Rev. Gastroenterol. Hepatol..

[7] Aslam N., Lo S.W., Sikafi R., Barnes T., Segal J., Smith P.J., Limdi J.K. (2022). A review of the therapeutic management of ulcerative colitis. Therap. Adv. Gastroenterol..

[8] Hirten R.P., Sands B.E. (2021). New Therapeutics for Ulcerative Colitis. Annu. Rev. Med..

[9] Chanez-Paredes S.D., Abtahi S., Zha J., Li E., Marsischky G., Zuo L., Grey M.J., He W., Turner J.R. (2024). Mechanisms underlying distinct subcellular localization and regulation of epithelial long myosin light-chain kinase splice variants. J. Biol. Chem..

[10] Graham W.V., He W., Marchiando A.M., Zha J., Singh G., Li H.S., Biswas A., Ong M., Jiang Z.H., Choi W. (2019). Intracellular MLCK1 diversion reverses barrier loss to restore mucosal homeostasis. Nat. Med..

[11] He W.Q., Wang J., Sheng J.Y., Zha J.M., Graham W.V., Turner J.R. (2020). Contributions of Myosin Light Chain Kinase to Regulation of Epithelial Paracellular Permeability and Mucosal Homeostasis. Int. J. Mol. Sci..

[12] Miranda-Bautista J., Rodriguez-Feo J.A., Puerto M., Lopez-Cauce B., Lara J.M., Gonzalez-Novo R., Martin-Hernandez D., Ferreiro-Iglesias R., Banares R., Menchen L. (2021). Liver X Receptor Exerts Anti-Inflammatory Effects in Colonic Epithelial Cells via ABCA1 and Its Expression Is Decreased in Human and Experimental Inflammatory Bowel Disease. Inflamm. Bowel Dis..

[13] Pellegrino R., Imperio G., De Costanzo I., Izzo M., Landa F., Tambaro A., Gravina A.G., Federico A. (2025). Small Molecules in the Treatment of Acute Severe Ulcerative Colitis: A Review of Current Evidence. Pharmaceuticals.

[14] Gudjonsson T., Joelsson J.P., Arason A.J., Asbjarnarson A., Gardarsson F.R., Lehmann F., Teodorovic P., Ingthorsson S., Sigurdsson S., Valdimarsdottir B. (2025). A novel macrolide, EP395, with reduced antibacterial activity and an enhancing effect on respiratory epithelial barrier. Pulm. Pharmacol. Ther..

[15] Kricker J.A., Norris V., Page C., Parnham M.J. (2025). Effects of EP395, a novel macrolide, on acute neutrophilic airway inflammation. Pulm. Pharmacol. Ther..

[16] Gordon S., Daneshian M., Bouwstra J., Caloni F., Constant S., Davies D.E., Dandekar G., Guzman C.A., Fabian E., Haltner E. (2015). Non-animal models of epithelial barriers (skin, intestine and lung) in research, industrial applications and regulatory toxicology. ALTEX.

[17] Schnur S., Wahl V., Metz J.K., Gillmann J., Hans F., Rotermund K., Zah R.K., Bruck D.A., Schneider M., Hittinger M. (2022). Inflammatory bowel disease addressed by Caco-2 and monocyte-derived macrophages: An opportunity for an in vitro drug screening assay. In Vitro Models.

[18] Nguyen O.T.P., Misun P.M., Hierlemann A., Lohasz C. (2024). A Versatile Intestine-on-Chip System for Deciphering the Immunopathogenesis of Inflammatory Bowel Disease. Adv. Healthc. Mater..

[19] Richter C., Latta L., Harig D., Carius P., Stucki J.D., Hobi N., Hugi A., Schumacher P., Krebs T., Gamrekeli A. (2025). A stretchable human lung-on-chip model of alveolar inflammation for evaluating anti-inflammatory drug response. Bioeng. Transl. Med..

[20] Low D., Nguyen D.D., Mizoguchi E. (2013). Animal models of ulcerative colitis and their application in drug research. Drug Des. Devel Ther..

[21] Yang C., Merlin D. (2024). Unveiling Colitis: A Journey through the Dextran Sodium Sulfate-induced Model. Inflamm. Bowel Dis..

[22] Sengupta A., Dorn A., Jamshidi M., Schwob M., Hassan W., De Maddalena L.L., Hugi A., Stucki A.O., Dorn P., Marti T.M. (2023). A multiplex inhalation platform to model in situ like aerosol delivery in a breathing lung-on-chip. Front. Pharmacol..

[23] Arason A.J., Joelsson J.P., Valdimarsdottir B., Sigurdsson S., Gudjonsson A., Halldorsson S., Johannsson F., Rolfsson O., Lehmann F., Ingthorsson S. (2019). Azithromycin induces epidermal differentiation and multivesicular bodies in airway epithelia. Respir. Res..

[24] Joelsson J.P., Kricker J.A., Arason A.J., Sigurdsson S., Valdimarsdottir B., Gardarsson F.R., Page C.P., Lehmann F., Gudjonsson T., Ingthorsson S. (2020). Azithromycin ameliorates sulfur dioxide-induced airway epithelial damage and inflammatory responses. Respir. Res..

[25] Miyagawa T., Fujita T., Yumoto H., Yoshimoto T., Kajiya M., Ouhara K., Matsuda S., Shiba H., Matsuo T., Kurihara H. (2016). Azithromycin recovers reductions in barrier function in human gingival epithelial cells stimulated with tumor necrosis factor-alpha. Arch. Oral Biol..

[26] Slater M., Torr E., Harrison T., Forrester D., Knox A., Shaw D., Sayers I. (2016). The differential effects of azithromycin on the airway epithelium in vitro and in vivo. Physiol. Rep..

[27] Elkholy S.E., Maher S.A., Abd El-Hamid N.R., Elsayed H.A., Hassan W.A., Abdelmaogood A.K.K., Hussein S.M., Jaremko M., Alshawwa S.Z., Alharbi H.M. (2023). The immunomodulatory effects of probiotics and azithromycin in dextran sodium sulfate-induced ulcerative colitis in rats via TLR4-NF-kappaB and p38-MAPK pathway. Biomed. Pharmacother..

[28] Mahgoub A., El-Medany A., Mustafa A., Arafah M., Moursi M. (2005). Azithromycin and erythromycin ameliorate the extent of colonic damage induced by acetic acid in rats. Toxicol. Appl. Pharmacol..

[29] Pan F., Han L., Zhang Y., Yu Y., Liu J. (2015). Optimization of Caco-2 and HT29 co-culture in vitro cell models for permeability studies. Int. J. Food Sci. Nutr..

[30] Reale O., Huguet A., Fessard V. (2020). Co-culture model of Caco-2/HT29-MTX cells: A promising tool for investigation of phycotoxins toxicity on the intestinal barrier. Chemosphere.

[31] Hoffmann P., Burmester M., Langeheine M., Brehm R., Empl M.T., Seeger B., Breves G. (2021). Caco-2/HT29-MTX co-cultured cells as a model for studying physiological properties and toxin-induced effects on intestinal cells. PLoS ONE.

[32] FDA (2025). FDA Announces Plan to Phase Out Animal Testing Requirement for Monoclonal Antibodies and Other Drugs.

[33] Marrer-Berger E., Nicastri A., Augustin A., Kramar V., Liao H., Hanisch L.J., Carpy A., Weinzierl T., Durr E., Schaub N. (2024). The physiological interactome of TCR-like antibody therapeutics in human tissues. Nat. Commun..

[34] Rumsey J.W., Lorance C., Jackson M., Sasserath T., McAleer C.W., Long C.J., Goswami A., Russo M.A., Raja S.M., Gable K.L. (2022). Classical Complement Pathway Inhibition in a “Human-On-A-Chip” Model of Autoimmune Demyelinating Neuropathies. Adv. Ther..

[35] LaFollette M.R., Baran S.W., Curley J.L., Dickinson A.M., Frazier T., Hobi N., Huang M.I., Hutter V., Maisonneuve B.G.C., Marsh G.A. (2025). The Use of MPS in Three Rs and Regulatory Applications: Perspectives From Developers on Stakeholder Responsibilities. Altern. Lab. Anim..

[36] Rimmer C., Hetelekides S., Eliseeva S.I., Georas S.N., Veazey J.M. (2024). Correction: Budesonide promotes airway epithelial barrier integrity following double-stranded RNA challenge. PLoS ONE.

[37] Sekiyama A., Gon Y., Terakado M., Takeshita I., Kozu Y., Maruoka S., Matsumoto K., Hashimoto S. (2012). Glucocorticoids enhance airway epithelial barrier integrity. Int. Immunopharmacol..

[38] Anderson S.J., Lockhart J.S., Estaki M., Quin C., Hirota S.A., Alston L., Buret A.G., Hancock T.M., Petri B., Gibson D.L. (2019). Effects of Azithromycin on Behavior, Pathologic Signs, and Changes in Cytokines, Chemokines, and Neutrophil Migration in C57BL/6 Mice Exposed to Dextran Sulfate Sodium. Comp. Med..

[39] Verburgt C.M., Heutink W.P., Kuilboer L.I.M., Dickmann J.D., van Etten-Jamaludin F.S., Benninga M.A., de Jonge W.J., Van Limbergen J.E., Tabbers M.M. (2021). Antibiotics in pediatric inflammatory bowel diseases: A systematic review. Expert. Rev. Gastroenterol. Hepatol..

