# Supplementary material for: In Vitro and In Vivo Efficacy of Epithelial Barrier-Promoting Barriolides as Potential Therapy for Ulcerative Colitis
Source: Biomedicines. 2026 Jan 21;14(1):237. doi: 10.3390/biomedicines14010237 (PMC12838928; doi:10.3390/biomedicines14010237)
Supplement: Supplementary file 1 [file biomedicines-14-00237-s001.zip › biomedicines-4047326-supplementary.pdf]

Supplementary Materials Figure S1

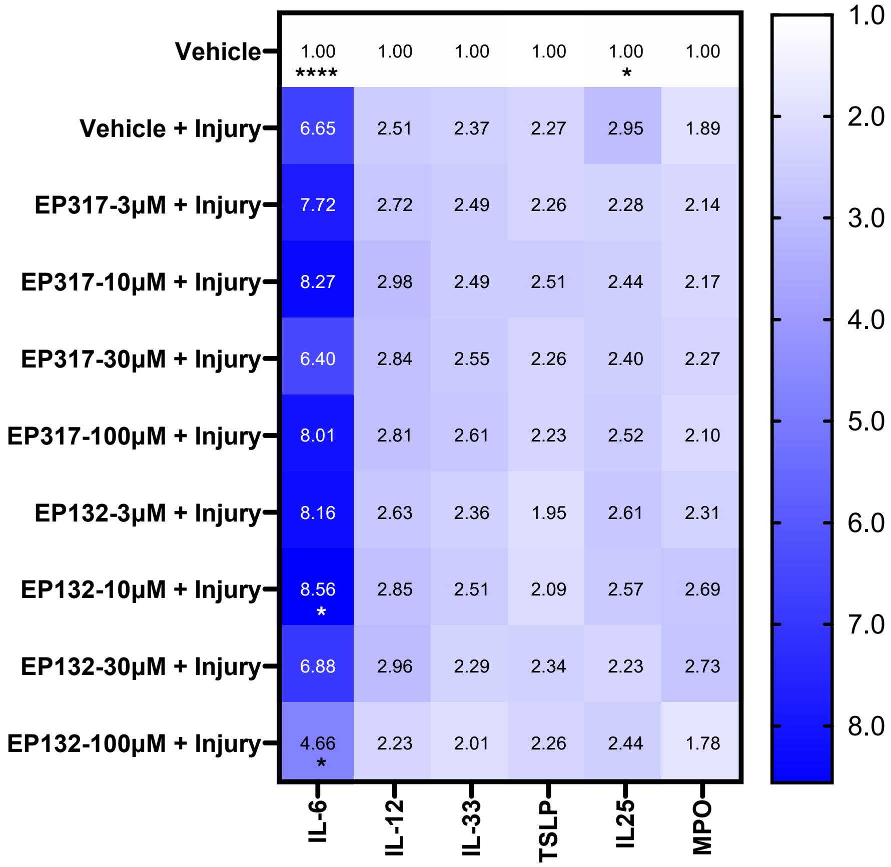

Supplementary Materials Figure S1. Effects of EP132 and EP317 on cytokine levels after epithelial injury. The cells in the ^{AX}Gut-on-Chip injury model were exposed to a cytokine challenge (injury) during barriolide treatment. The medium supernatant was sampled apically at the endpoint and stored at -80ºC. All samples were analysed using the Human Luminex Discovery Assay (LXSAHM-07, R&D Systems, Minneapolis, MN, USA). Values are shown relative to the vehicle group average fluorescence intensity. Samples are from a single experiment but measured in replicates (n=4-6) and were analysed using Dunnett's multiple comparisons test, comparison to Vehicle+ Injury * $p < 0.05$, **** $p < 0.0001$.
